# Substantial Hierarchical Reductions of Genetic and Morphological Traits in the Evolution of Rotiferan Parasites

**DOI:** 10.1093/gbe/evaf124

**Published:** 2025-06-19

**Authors:** Holger Herlyn, Anju Angelina Hembrom, Juan-Pablo Tosar, Katharina M Mauer, Hanno Schmidt, Bahram Sayyaf Dezfuli, Thomas Hankeln, Lutz Bachmann, Peter Sarkies, Kevin J Peterson, Bastian Fromm

**Affiliations:** Institute of Organismic and Molecular Evolution, Johannes Gutenberg University Mainz, Mainz, Germany; The Arctic University Museum of Norway, UiT—The Arctic University of Norway, Tromsø, Norway; Functional Genomics Laboratory, Institut Pasteur de Montevideo, Montevideo, Uruguay; Analytical Biochemistry Unit, Center for Nuclear Research, School of Science, Universidad de la República, Montevideo, Uruguay; Institute of Organismic and Molecular Evolution, Johannes Gutenberg University Mainz, Mainz, Germany; Institute of Organismic and Molecular Evolution, Johannes Gutenberg University Mainz, Mainz, Germany; Institute of Organismic and Molecular Evolution, Johannes Gutenberg University Mainz, Mainz, Germany; Department of Life Sciences and Biotechnology, University of Ferrara, Ferrara, Italy; Institute of Organismic and Molecular Evolution, Johannes Gutenberg University Mainz, Mainz, Germany; Natural History Museum, University of Oslo, Oslo, Norway; Department of Biochemistry, University of Oxford, Oxford, UK; Department of Biological Sciences, Dartmouth College, Hanover, NH, USA; The Arctic University Museum of Norway, UiT—The Arctic University of Norway, Tromsø, Norway

**Keywords:** complexity, microRNAs, piRNAs, core genes, Rotifera, evolution

## Abstract

Within the last 800 million years, animals evolved a vast range of diversity of species exhibiting an enormous disparity of forms and lifestyles. The process involved an increase in complexity from life forms with few cell types to organisms with many hundreds of cell types. However, neither genome size nor number of protein-coding genes can explain these differences, and their biological basis remains elusive. Yet, recent studies suggest that the evolution of complexity is closely linked to the acquisition of a class of noncoding gene regulators called microRNAs. To test this hypothesis, we investigated the association between loss of organismal complexity and microRNAs in Syndermata, an invertebrate group including free-living wheel animals (Monogononta, Bdelloidea), epibiotic Seisonidea, and endoparasitic thorny-headed worms (Acanthocephala). Analyses of genomic, transcriptomic, and morphological data of altogether 25 syndermatan species revealed strong correlations of microRNA losses with reductions of protein-coding genes and morphological traits. The hierarchical pattern sums up to ∼85% loss of microRNAs and a ∼50% loss of conserved metazoan core genes (Benchmarking Universal Single-Copy Orthologs) on the lineage to thorny-headed worms. Extraordinarily reduced microRNA complements were confirmed by small RNA sequencing data. Endoparasitic Acanthocephala was additionally distinguished by the most morphological reductions of ancestral features, such as the digestive tract. Together, we observed that reductions of ∼400 protein-coding genes and 10 metazoan core genes tended to accompany the loss of single microRNA families. Furthermore, 4 microRNA families and 34 metazoan core genes appeared to be associated, on average, with the reduction of a single morphological trait.

## Graphical Abstract

**Graphical Abstract:**
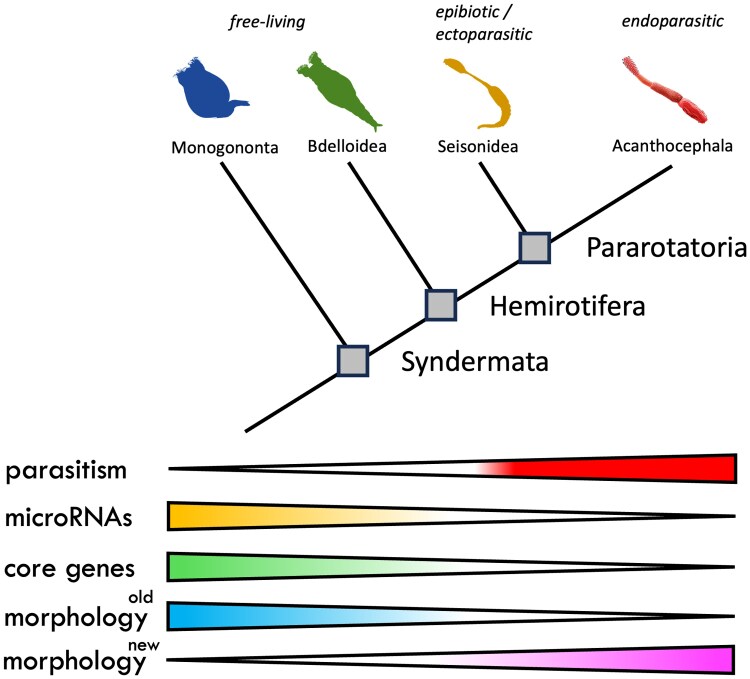

Significance Over 800 million years, animal complexity increased from simple to highly diverse and complex organisms. However, genome size and protein-coding genes do not explain this increase in complexity. Recent studies link the evolution of complexity to microRNAs, key regulators of gene translation. This study explored the loss of microRNAs in Syndermata, including free-living rotifers and parasitic Acanthocephala. It revealed a strong correlation between stepwise reduction of organismal complexity, significant loss of microRNAs (up to 85%), and protein-coding genes. The hierarchical loss of microRNAs paralleled the loss of morphological features and core genes. These findings support a potential role for microRNAs in maintaining organismal complexity and suggest that their loss is linked to genomic and morphological simplifications observed during the evolution of parasitism.

## Introduction

The evolution from life forms with very few cell types to complex organisms with many cell types, tissues and organs, and high cognitive abilities must have a genetic basis (Valentine et al. 1994). However, the underpinnings of the complexity–genome relation remain to be clarified, since neither genome size (Cavalier-Smith 1978; Gregory 2001) nor protein-coding gene number (Hahn and Wray 2002) is indicative of animal complexity (C- and G-value paradox, respectively). In fact, the molecular basis of the large divergence in organismic complexity remains one of the biggest mysteries in biology.

Recent studies suggest that the evolution of novel cell and tissue types is hierarchical and tightly correlated with the acquisition of microRNAs (Sempere et al. 2006; Tanzer and Stadler 2006; Grimson et al. 2008; Heimberg et al. 2008; Peterson et al. 2009; Wheeler et al. 2009; Christodoulou et al. 2010; Berezikov 2011; Fromm et al. 2015; Alberti et al. 2018; Deline et al. 2018; Zolotarov et al. 2022; Fromm 2024; Clarke et al. 2025). MicroRNAs are ∼22 nucleotide short noncoding gene regulators involved in a plethora of biological processes (Bartel 2018). By regulating messenger RNA translation, either in a switch-like or rheostat manner, microRNAs can diversify existing genetic programs and can canalize the evolution of new cell types and phenotypes (Hornstein and Shomron 2006; Peterson et al. 2009; Wu et al. 2009; Avital et al. 2018; Chakraborty et al. 2020). The high developmental relevance of microRNAs is reflected in deep evolutionary conservation and rare losses of their genes from genomes (Pasquinelli et al. 2000; Lagos-Quintana et al. 2001; Lau et al. 2001; Lee and Ambros 2001; Sempere et al. 2006; Wheeler et al. 2009). Correspondingly, they are excellent phylogenetic and taxonomic markers (Wheeler et al. 2009; Helm et al. 2012; Tarver et al. 2013, 2018; Kenny et al. 2015; Fromm et al. 2019; Fromm 2024) down to the species level (Fromm et al. 2014; Ovchinnikov et al. 2017; Kang et al. 2018). However, signals of microRNA-coupled increases of animal complexity can be obscured by great evolutionary timescales, the lack of annotated microRNAs in important groups and the challenge of determining accurate cell type numbers and their homologization in diverse animal clade (but see Zolotarov et al. 2022; Jenike et al. 2023).

Experimental approaches such as deleting or copying individual microRNAs are possible, but recreating macroevolutionary changes like increases or decreases in cellular complexity are not feasible as the required genetic changes, such as the deletion of whole families, generally lead to lethality (Alvarez-Saavedra and Horvitz 2010). However, evolutionary changes in complexity can also involve reductions of morphology and the loss of developmental stages or whole organs. Those are known to occur especially in microscopic, sessile, and parasitic species, and hence, the study of parasites holds an opportunity to shed light on the connection of microRNAs, protein-coding genes, and animal complexity.

Parasitism has evolved more than 200 times independently in animals since as early as the Cambrian (Weinstein and Kuris 2016; Cong et al. 2017) and significantly contributes to extant diversity. There are an estimated ∼300,000 parasitic helminth species alone, infecting nearly all of the ∼79,000 known vertebrate species (Dobson et al. 2008; Parr et al. 2014). Parasites are recognized for substantial changes in morphology enabling life on or inside hosts, such as losses of whole organs or the emergence of new structures for attachment (Poulin 2011). More recently, it has become clear that parasite evolution is often reflected in genome simplification, including large losses of genes and regulatory elements. However, it is highly debated whether morphological reductions in parasite evolution go along with loss of genomic features (Tsai et al. 2013; Hahn et al. 2014; Zarowiecki and Berriman 2015; International Helminth Genomes Consortium 2019) (and see Jackson 2015; Adams et al. 2020).

We addressed whether there is a connection between the reductions of morphological traits, protein-coding genes, and microRNAs in rotifers including their parasitic representatives (Syndermata AHLRICHS, 1995). The protostome group is composed of microscopic “wheel animals” (Rotifera CUVIER, 1817) belonging to either Monogononta PLATE, 1889 or Bdelloidea HUDSON, 1884, as well as Pararotatoria (Sielaff et al. 2016; Vasilikopoulos et al. 2024). The latter includes Seisonidea WESENBERG-LUND, 1899 measuring in the millimeter range and macroscopic “thorny-headed worms” or Acanthocephala KOELREUTER, 1771. Most monogononts and bdelloids are free-living aquatic organisms, while there are only few parasites in both taxa (May 1989). Moreover, marine seisonids live strictly on leptostracan crustaceans, potentially as ectoparasites (Plate 1887). Lastly, endoparasitic acanthocephalans have conquered almost every habitat by exploitation of mandibulates as intermediate and gnathostome vertebrates as definitive hosts. Acanthocephalans lack a digestive tract and take up nutrients via a modified tegument in all developmental stages. Adults are further endowed with an elaborated attachment organ at the anterior body pole (Meyer 1933; Herlyn 2021).

We analyzed syndermatans for their microRNA complements, protein-coding gene repertoires, morphological traits, and lifestyles. For this purpose, we generated the first small RNA sequencing datasets for acanthocephalans and seisonids. Together with publicly available genome and small RNA sequencing data, we annotated microRNA complements of 25 syndermatan species including 11 monogonont and 11 bdelloid rotifers, in addition to one seisonid and two acanthocephalan species. We further compared protein-coding gene repertoires and single-copy protein-coding core genes, which have been postulated for the last common metazoan ancestor due to their wide conservation in descendent lineages using Benchmarking Universal Single-Copy Orthologs (BUSCO) (Manni et al. 2021). Our analyses additionally incorporated an extended morphological character and lifestyle matrix built upon previously published data (e.g. Deline et al. 2018). Within Syndermata, we observed an apparent hierarchical loss of microRNAs in the lineage to endoparasitic acanthocephalans, with 25% of the expected microRNA families missing in monogononts and bdelloids, and an unprecedented overall loss of usually conserved microRNA families in the seisonid and acanthocephalans (up to potentially 67% and 85%, respectively). This substantial loss of microRNAs was highly correlated with likewise strong reductions in protein-coding gene numbers and metazoan core gene complements. Losses of microRNAs and protein-coding genes further correlated strongly with numbers of morphological reductions. Focusing on Syndermata, this study underlines that microRNA evolution is closely intertwined with morphological trait and lifestyle evolution.

## Results

### The MicroRNA Complements of Syndermatans Show Substantial Hierarchical Losses

**Fig. 1.**
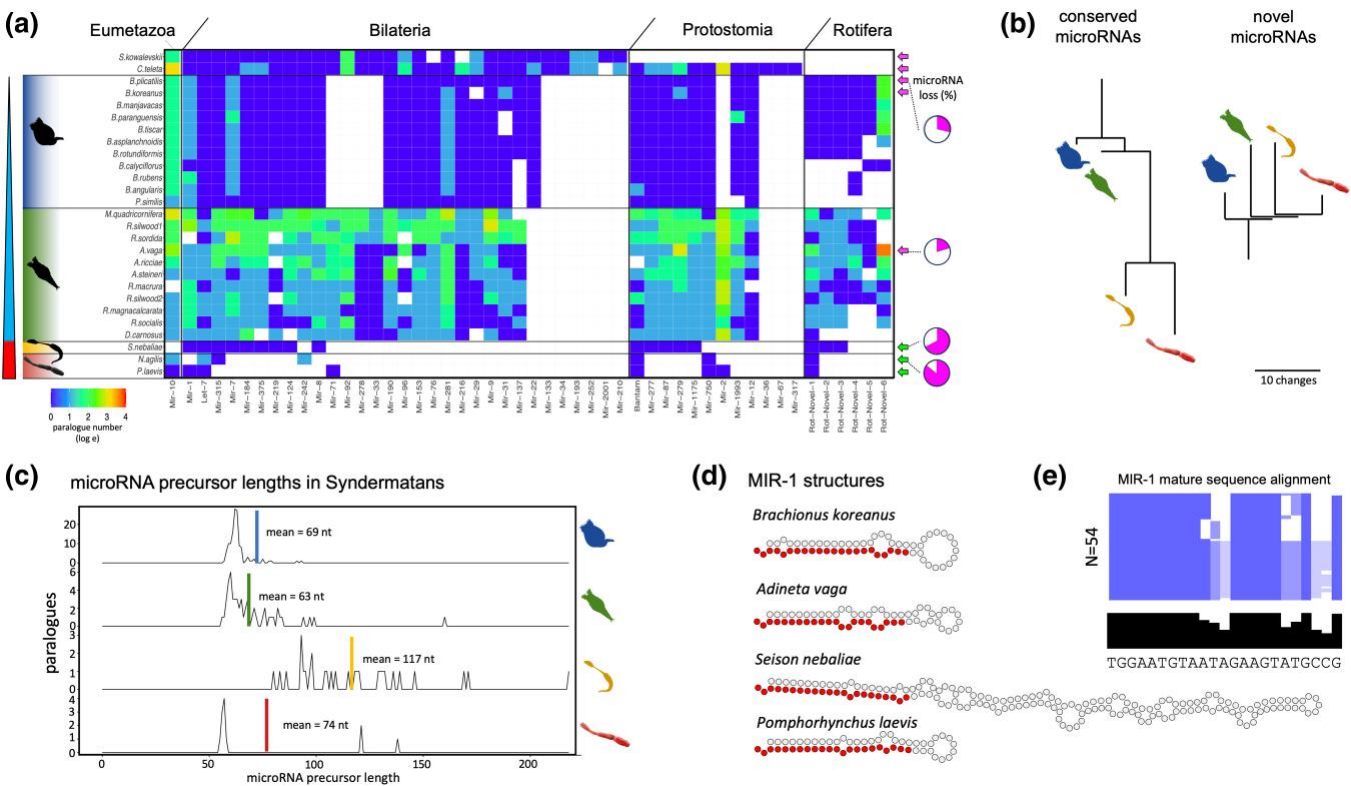
MicroRNA complements of Syndermata reflect stepwise losses from free-living to parasitic ancestors and unprecedented losses of 85% of conserved microRNAs in acanthocephalans. a) Banner-plot of 25 microRNA complements of included syndermatan species and two outgroups. White field means that no microRNA was found. Heatmap function refers to the paralogue number in each microRNA family (log e). Note that both outgroups do not belong to Syndermata and, hence, have none of the Rotifera microRNA families. Furthermore, *S. kowalevskii* is not a protostome and hence lacks protostome microRNA families. See supplementary file S1, Supplementary Material online “species microRNA families”. Arrows depict availability of small RNA sequencing data (pink, publicly available; green, novel). b) Schematic trees of the four syndermatan groups representing loss of usually conserved and gain of novel microRNA families. Branch lengths correspond to the number of gains and losses. c) Overview of microRNA precursor lengths in selected representatives of monogononts (*B. plicatilis*), bdelloids (*A. vaga*), the seisonid *S. nebaliae*, and the acanthocephalan *P. laevis*. d) Selected MIR-1 examples of the same species as in c) highlighting the length deviations in *S. nebaliae*. e) Alignment of the mature sequence of all MIR-1 genes in syndermatans (*N* = 54) highlights its very strong conservation in the pattern characterized for bilaterian mature miRNAs (Wheeler et al. 2009; Fromm et al. 2015) with strong conservation in the seed and 3′ CR and higher variability in positions 9 to 12 and 17 to 22. Species order corresponds to a); consensus is given in black.

Given the phylogenetic position of Syndermata within Metazoa (e.g. Struck et al. 2014; Bleidorn 2019; Laumer et al. 2019), 1 eumetazoan, 31 bilaterian, and 12 protostome microRNA gene families were expected to be conserved in the analyzed 25 species. However, significant numbers of the altogether 44 microRNA families were missing in the species studied (Fig. 1a and b, supplementary file S1, Supplementary Material online, “species microRNA families”) according to genome-based predictions by MirMachine (Umu et al. 2023). The losses amounted to a minimum of ten microRNA families in free-living wheel animals (Bdelloidea, 10 losses; Monogononta, 14 losses). The epibiotic representative of Seisonidea, *Seison nebaliae*, lacked 30 of the expected microRNA families, and 37 microRNA families were absent in endoparasitic Acanthocephala (Fig. 1a). Complementary analyses of small RNA sequencing datasets for representatives of each group with MirMiner (Wheeler et al. 2009) confirmed the losses of the conserved microRNA families and identified six additional shared microRNA families in the monogonont and bdelloid representatives. Of these, three and one were also identified in the seisonid and the acanthocephalan species, respectively (Fig. 1a). Given the phylogenetic relationships within the group (Sielaff et al. 2016; Vasilikopoulos et al. 2024; Luo et al. 2025), the six genes probably emerged in the syndermatan stem line, were retained in most monogononts and bdelloids, and got partially lost in pararotatorian evolution. For the other microRNA families, MirMiner confirmed paralogue numbers as predicted by MirMachine. This included the identification of additional gene copies for most microRNA families in *Adineta vaga*, reflecting genome duplication in the bdelloid stem line (Simion et al. 2021). Together, MirMachine and MirMiner annotations recognized 54 microRNAs for the monogonont *Brachionus plicatilis* (35 conserved families, https://mirgenedb.org/browse/bpl), 129 microRNAs for the bdelloid *A. vaga* (39 conserved families, https://mirgenedb.org/browse/ava), and only 29 microRNAs for the seisonid *S. nebaliae* (16 conserved families, https://mirgenedb.org/browse/sne). Even fewer microRNAs were identified in the acanthocephalans, with 15 (seven conserved families) in the eoacanthocephalan *Neoechinorhynchus agilis* (https://mirgenedb.org/browse/nag) and 12 (seven conserved families) in the palaeacanthocephalan *Pomphorhynchus laevis* (https://mirgenedb.org/browse/ple). All microRNAs were detected in the respective genomes and small RNA sequencing data, respectively. See supplementary fig. S1, Supplementary Material online for details on the six microRNA sequencing datasets produced herein for *Seison* and the two acanthocephalans. MicroRNA annotations, expression data, and genomic locations are available for the species with small RNA sequencing data on MirGeneDB (Clarke et al. 2025). In line with the expectation of a stepwise decrease of microRNA complements with growing ties to a host, microRNA family losses amounted up to 20% in bdelloids and 29% in monogononts, and potentially up to 67% in the seisonid and 85% in the acanthocephalans, respectively. Notably, detailed analyses of small RNA sequencing did not uncover any reads of the missing microRNA families, neither in the seisonid nor in the acanthocephalans. To test for a detection bias for highly expressed microRNAs, expression levels of the detected microRNAs in pararotatorians were further compared to the free-living relatives and outgroup representatives and no pattern was found (supplementary file S1, Supplementary Material online, “microRNA expression”). Thus, absence of so many microRNAs is unlikely to be due to false negatives caused by limited genome assembly qualities, as supported by multiple independent lines of evidence including small RNA-Seq data and high mRNA mapping rates. Instead, the observation is suggestive of hierarchical and progressive reduction of microRNA repertoires on the lineage to Acanthocephala (Fig. 1b). In fact, loss levels encountered in thorny-headed worms are the highest reported for any bilaterian animal to date. MirMiner additionally predicted several novel species-specific microRNAs (Fig. 1b).

Furthermore, novel and conserved microRNAs displayed expected hairpin lengths of mean of 69 nucleotides in monogononts, 63 nucleotides in bdelloids, and 59 to 74 nucleotides in acanthocephalans. Contrary to this, the seisonid *S. nebaliae* was distinguished by remarkably long hairpins of around 75 to 175 nucleotides (mean of 117 nucleotides). A single hairpin even exceeded 200 nucleotides in this species. Despite dramatic differences in the length of pre-microRNAs, mature sequences showed the expected high level of conservation (Tarver et al. 2013) in all syndermatan members, as exemplified in MIR-1 (Fig. 1d and e). Similarly, long hairpins were reported before in parasitic flatworms (Fromm et al. 2013).

### Hierarchical Loss of Protein-Coding Genes Reflects MicroRNA Losses

**Fig. 2.**
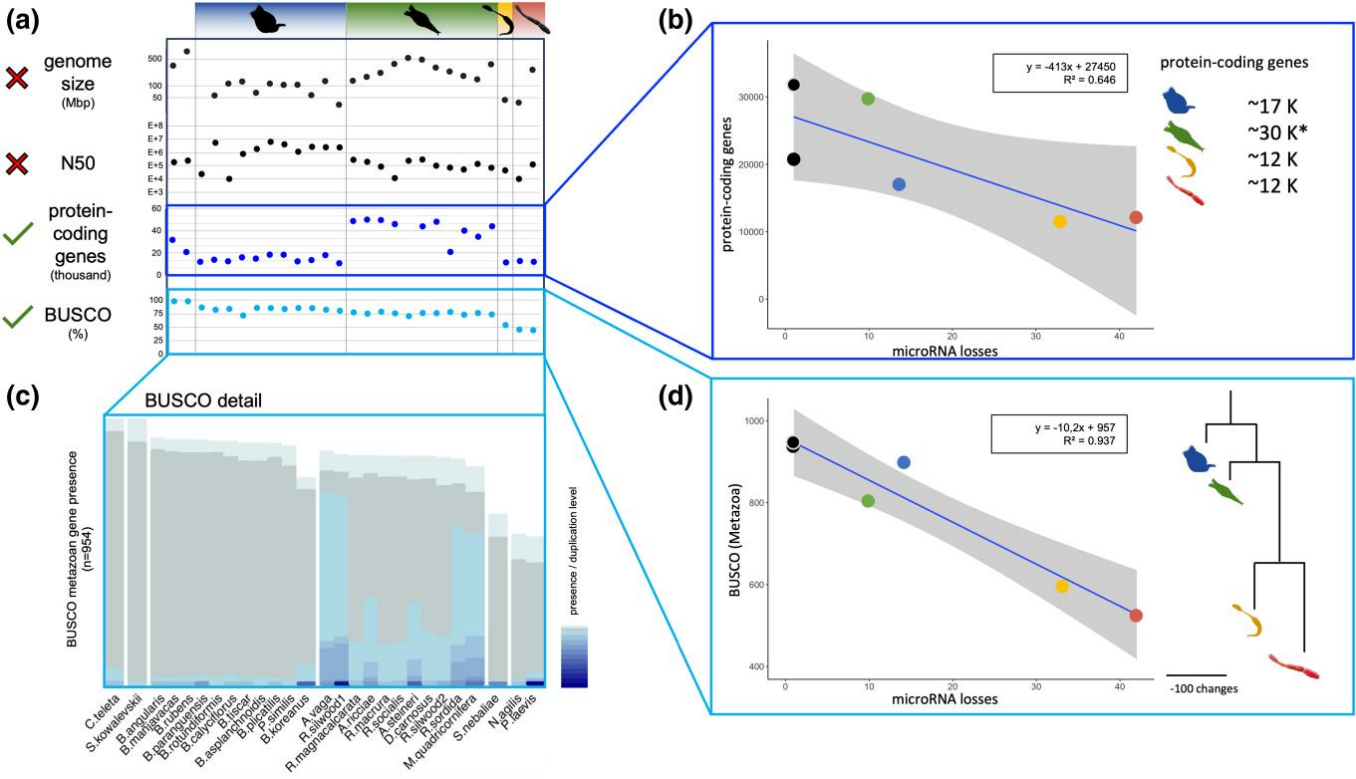
Numbers of protein-coding genes and protein-coding core genes (BUSCO), but not genome size or assembly quality (N50), correlate with microRNA loss. a) Genome size, N50, number of protein-coding genes, and metazoan BUSCO completeness of 27 syndermatan species and two outgroup representatives (see supplementary file S1, Supplementary Material online “Syndermata genomes”). b) Correlation of protein-coding gene numbers with cumulative numbers of microRNA losses in representative syndermatans (*B. koreanus*, *A. vaga*, *S. nebaliae*, *P. laevis*) and two outgroup representatives (*C. teleta*, *S. kowalevski*). Gray area corresponds to 95% confidence interval. The asterisk (*) refers to a high number of paralogues in bdelloids due to genome duplication (see supplementary file S1, Supplementary Material online “correlations”). c) BUSCO completeness highlights duplicated genomes in bdelloids (see supplementary file S1, Supplementary Material online “Busco_syndermatan”). d) Correlation of metazoan BUSCO core genes with the loss of microRNA genes in the same representative syndermatans as in b) and tree giving BUSCO gene losses. Gray area in graph corresponds to 95% confidence interval. Branch lengths correspond to the number of losses.

To test whether the extensive reductions of syndermatan microRNA complements were reflected in other features of their genomes, we compared genome size estimates, genome assembly contiguity (N50), and numbers of annotated protein-coding genes and protein-coding core genes (BUSCO) (Manni et al. 2021). Low correlation coefficient values revealed that genome size estimates or assembly quality could not explain microRNA losses (Fig. 2a, R^2^ = 0.17 and R^2^ = 0.08, respectively, supplementary file S1, Supplementary Material online “correlations”, supplementary file S1, Supplementary Material online “syndermatan genomes”), reinforcing the above notion that false negatives are unlikely to account for the cumulative loss of microRNAs on the lineage to parasitic acanthocephalans. Instead, we observed a tight association between numbers of protein-coding genes (as taken from Hagemann et al. 2024) and microRNA families. On average, 424 protein-coding genes went lost per missing microRNA family (R^2^ = 0.64) (Fig. 2b, supplementary file S1, Supplementary Material online “correlations”). This suggests a potential link between the reduction of microRNA families and a reduction of protein-coding genes. To further elaborate on this, we focused on 954 protein-coding genes with clear orthology relations that have been postulated for the last common ancestor of metazoans (BUSCO: Metazoa node (Manni et al. 2021)). By doing so, we observed a tight correlation of microRNA and metazoan core gene losses (R^2^ = 0.93; Fig. 2d, supplementary file S1, Supplementary Material online “correlations”), with 11 BUSCO genes lost per microRNA family reduced. Considering the phylogenetic relationships within Syndermata, this suggests a hierarchical and progressive reduction of universal single-copy metazoan orthologs from free-living to epibiotic and endoparasitic ancestors (Fig. 2c and d, supplementary file S1, Supplementary Material online “BUSCO_Syndermatans”). Thus, we observed 54 shared metazoan core gene losses in all syndermatans and additional 61 and 165 shared losses in Hemirotifera (Bdelloidea plus Pararotatoria) and Pararotatoria (Seisonidea plus Acanthocephala), respectively. *Seison nebaliae* lacked 76 and the Acanthocephala 148 further BUSCO genes in addition.

### Strong Enrichment of Gene Regulation Ontologies in the Lost BUSCO Genes

**Fig. 3.**
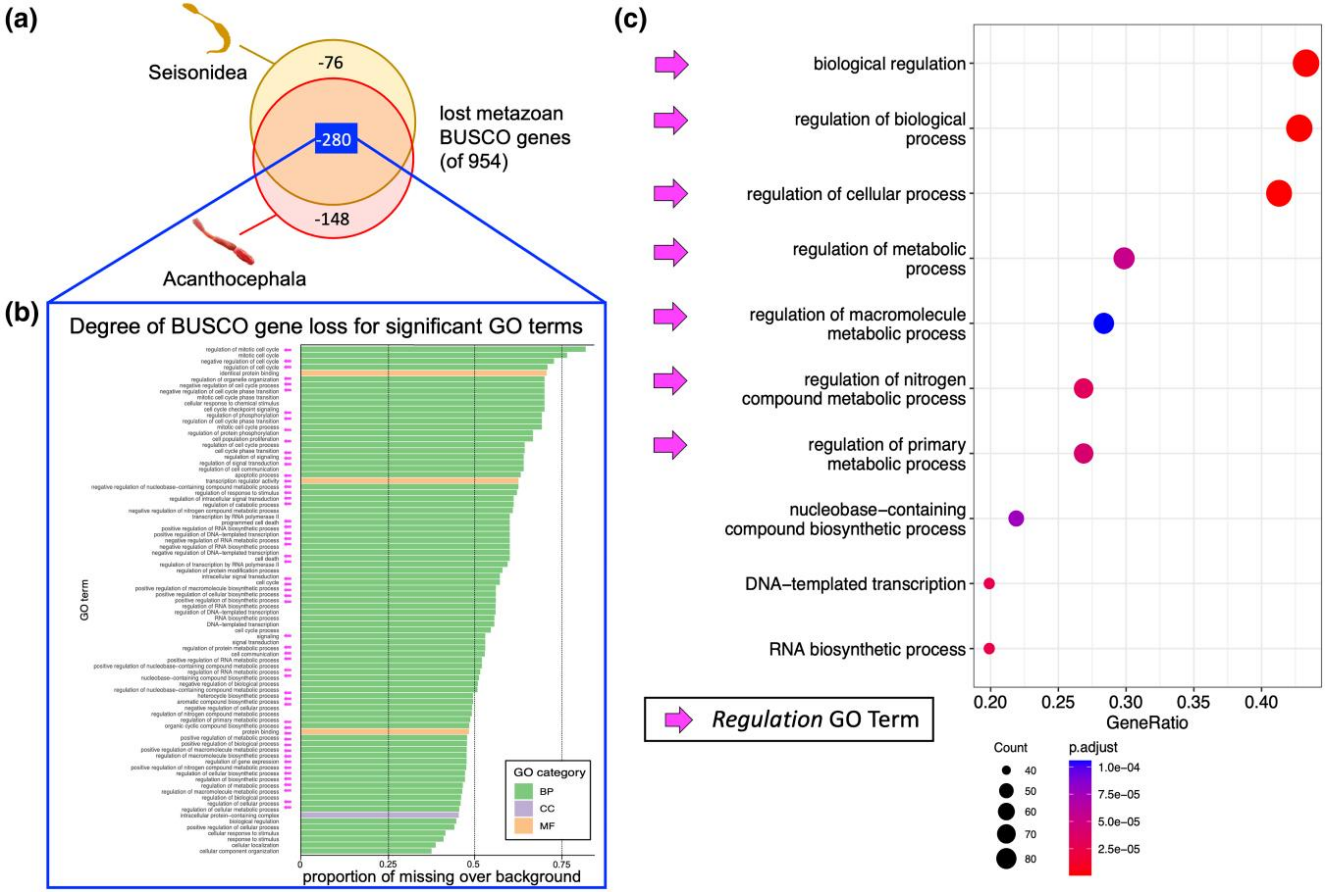
Missing BUSCO genes in Pararotatoria are highly enriched for biological regulation GO terms. a) Venn diagram illustrating the overlap of missing metazoan BUSCO genes in the Pararotaria representatives *S. nebaliae* (Seisonidea) and *Neoechinorhynchus agilis* (Acanthocephala). Of the 954 protein-coding genes usually conserved in Metazoa, the seisonid and acanthocephalan collectively lack 280 genes. Additional 76 and 148 genes are absent in either the seisonidean or the acanthocephalan, respectively. b) Significantly enriched GO terms in the metazoan BUSCO genes missing in Pararotatoria. Enrichment of three *Molecular Function* GOs and one *Cellular Component* GO contrast with 81 enriched *Biological Process* GO terms. Note the high number of terms referring to regulation (pink arrows). c) Significantly enriched *Biological Process* GOs for missing genes in pararotatorians, whereby each dot represents one term. Color and weight of the dots correspond to the scale below the plot. Note the high number of regulatory terms (pink arrows).

Subsequently, we addressed the functional implications of extreme gene loss related to the transition from free-living to a host-bound living in the pararotatorian stem line. For this purpose, we conducted enrichment analyses of Gene Ontology (GO) terms in the 280 BUSCO genes missing in *S. nebaliae* and both acanthocephalans (Fig. 3a, supplementary file S2, Supplementary Material online). The lacking genes were significantly enriched for terms of all three major categories, i.e. *Biological Process*, *Cellular Component*, and *Molecular Function* (Fig. 3b). For *Cellular Component* and *Molecular Function*, we observed one (intracellular protein-containing complex) and three (protein binding, identical protein binding, transcription regulator activity) enriched annotations, respectively. Within the *Biological Process* category, we identified 81 enriched GO terms. Of those, the majority were associated with regulatory terms such as *regulation of biological process*, *biological regulation*, and *regulation of cellular process* (Fig. 3b and c, pink arrows). Consultation of the GeneCards database uncovered that more than 50% of the genes missing in Pararotatoria would, if present, code for positive effectors of transcription (such as transcription factor 25, mediator of RNA Pol II subunits—MED19/10/8/7/20), transcription repressors (such as negative elongation factor—NELFB/C/D), and other DNA-binding factors. The encoded proteins were components of RNA binding proteins and mediator complexes implicated in the basal RNA Pol II transcription machinery. We also observed involvements in cell division and proliferation as exemplified by Cyclin-Q and Cyclin-H. Genes implicated in the regulation of *Initiation of protein synthesis* like eukaryotic translation initiation factors 2 (eIF2 subunits D, B1, B2, B3, B5) and 3 (eIF3 subunits K, L, G, M) were additionally absent in pararotatorians.

### Morphological Losses Retrace Gene and Gene Regulator Losses

**Fig. 4.**
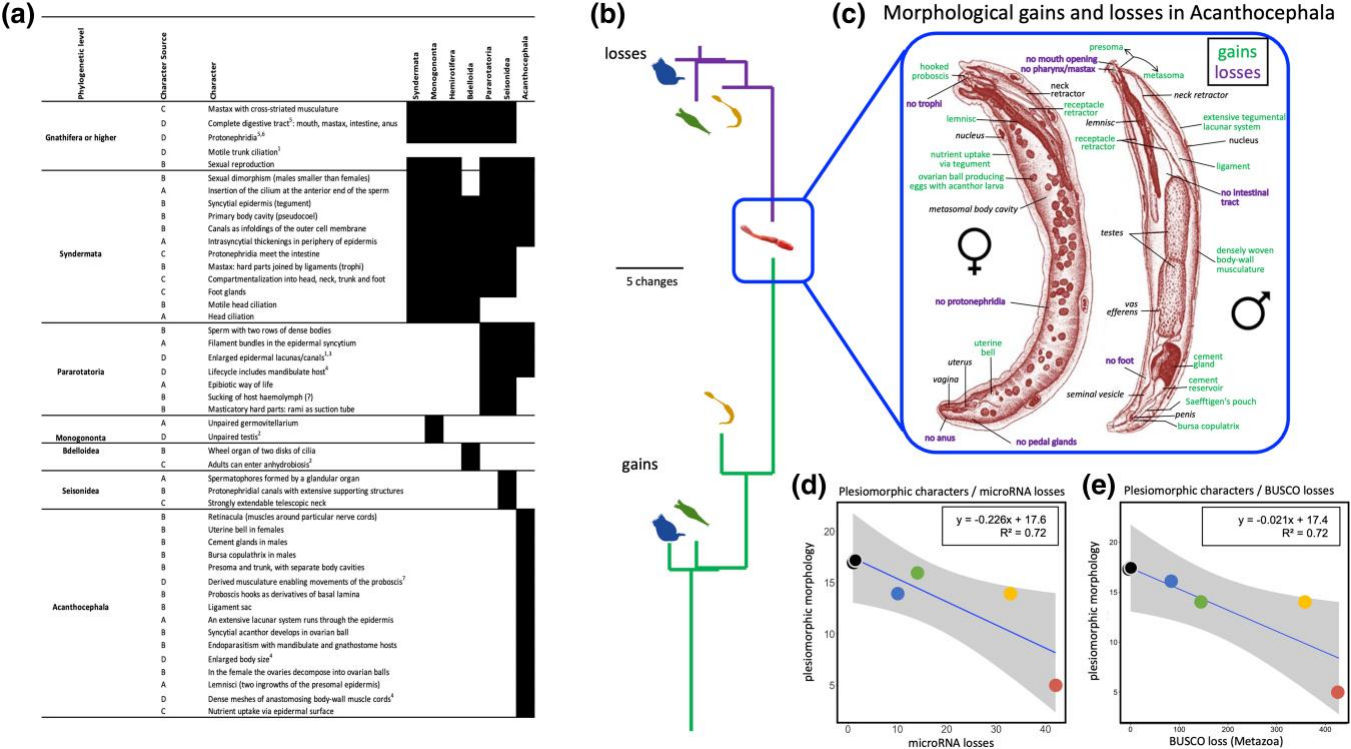
a) Syndermatan character matrix combining novel characters with the ones compiled by Deline et al. (2018). Dark gray versus white in taxon columns indicates character presence versus absence. Column 2 indicates the source of respective characters: A—Deline et al. (2018), B—modified wording of character definition by Deline et al. (2018), C—Ahlrichs (1995), D—assessment by authors considering: ^1^Ahlrichs (1997), ^2^Fontaneto and De Smet (2015), ^3^Nicholas and Mercer (1965), ^4^Herlyn (2021), ^5^Ahlrichs (1995), ^6^Amin (1987), ^7^Herlyn and Taraschewski (2017). b) Schematic trees of the four syndermatan groups representing loss of plesiomorphic (purple, top) and gain of apomorphic (green, bottom) morphological features. Branch lengths correspond to gains and losses. c) Morphological character loss (purple) and gain (green) in the extreme example of Acanthocephala. The eponymous attachment organ (proboscis) is inverted in the juvenile female and everted in the adult male. Drawings refer to *Neoechinorhynchus rutili*, a close relative of *N. agilis*. Modified from Steinsträsser (1936). d) Dependence of plesiomorphic morphological characters on loss of microRNA genes in representative syndermatans and two outgroup representatives (same species as in Fig. 2). e) Interrelations of metazoan BUSCO core genes and loss of plesiomorphic morphological characters in representative syndermatans and two outgroup representatives. Outgroup species are the same as in Fig. 2 and were set to zero morphological losses. The last common ancestor of extant Acanthocephala might still have possessed protonephridia. In any case, the two acanthocephalans studied (*P. laevis* and *N. agilis*) lack protonephridia.

To quantitatively assess morphological losses and gains, we expanded the list of syndermatan entries in the character matrix by Deline et al. (2018), which was derived from Ax (2012, 2013a, 2013b); see Materials and Methods. For a clear semantic distinction, we classified characters or states present in the last common ancestors of Gnathifera AHLRICHS, 1995 or Syndermata as plesiomorphies. In contrast, we refer to characters or states that should have arisen on individual branches of the syndermatan tree as to apomorphies, although the same states are formally represent plesiomorphies on descendent lineages. The resulting character matrix (Fig. 4a) led to trees giving losses of evolutionarily older traits (plesiomorphies) and apomorphy gains (Fig. 4b, supplementary file S1, Supplementary Material online, “morphology”). These illustrated the most extensive loss of plesiomorphic characters such as trunk and head ciliation, the total digestive system, and protonephridia on the lineage to both acanthocephalans after its split from the seisonid lineage (see Fig. 4c for details). Notably, branch length relations were highly similar in trees depicting losses of plesiomorphic characters (Fig. 4b), microRNA families (Fig. 1b), and metazoan core genes (Fig. 2b). In line with this, we observed strong associations of plesiomorphy and microRNA losses (Fig. 4d) on the one hand, and BUSCO gene and plesiomorphic character losses on the other (Fig. 4e) (R^2^ = 0.72 in both cases).

In summary, the loss of ∼400 protein-coding genes (Fig. 2b, R^2^ = 0.64) or ∼10 metazoan core genes (Fig. 2d, R^2^ = 0.93) correlates with one microRNA family loss; and the loss of ∼4 microRNA families or ∼34 metazoan core genes is associated with one lost plesiomorphic morphological feature. Partial correlation analysis underlined that the correlation between morphology losses und microRNA losses (coefficient = 0.905) was significant (*P* = 0.035) even if controlling for a potential effect of genome assembly contiguity (N50). Either way, morphological character gains, apomorphies, also occurred, and they exceeded amounts of losses on most branches. We noticed the strongest extent of character gains (absolute and relative to losses) for the acanthocephalan branch, followed by the branches to epibiotic seisonids and free-living rotifers. The detailed nature of these apomorphies (composition of cell types, or gene regulatory networks) remains to be studied, but is unlikely to be driven by the expansion of microRNAs and new cell types.

### Syndermatans Retain piRNAs

**Fig. 5.**
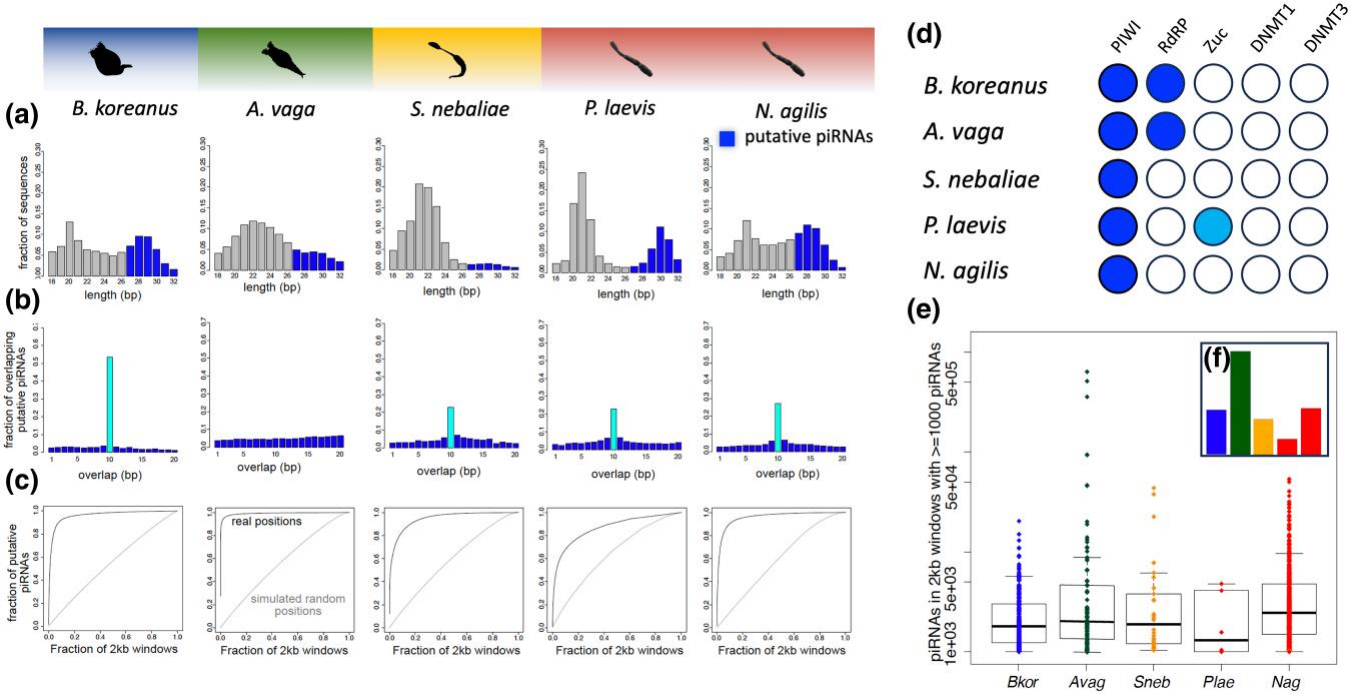
piRNA retention in syndermatans. a) Length distribution of genome-mapped small noncoding RNAs, with putative piRNAs highlighted. b) Distribution of overlap between putative piRNAs on opposite strands, highlighting a peak at 10nt where present, indicative of ping-pong biogenesis. c) Cumulative frequency plot showing the tendency of putative piRNAs to be clustered together along the genome, suggestive of potential piRNA-generating loci or piRNA clusters. d) Conservation of proteins related to small noncoding RNA pathways. Annotation of the presence or absence of homologue genes based on best reciprocal blast searches is illustrated for each species accordingly (dark blue, multiple hits; light blue, one hit). e) The piRNAs/2 kb windows for each of the 2 kb windows with at least 1,000 piRNAs mapped. f) The clustering ratio, defined as the ratio of the fraction of the genome containing 90% of randomly shuffled piRNAs compared to the fraction containing 90% of true piRNA positions. A higher clustering ratio thus indicates that a smaller fraction of the genome contains 90% of the piRNAs than expected by chance. *Avag*, *A. vaga*; *Bkor*, *B. koreanus*; *Nag*, *N. agilis*; *Plae*, *P. laevis*; *Sneb*, *S. nebaliae*.

Previously, loss of Piwi-interacting small RNAs (piRNAs) and DNA methylation was observed in parasitic flatworms and many parasitic nematodes (Tsai et al. 2013; Zheng 2013; Skinner et al. 2014; Sarkies et al. 2015; Fontenla et al. 2021; Sarkies 2022). The loss of piRNAs, in particular, was suggested to be connected to reduced complexity in parasites (Tsai et al. 2013; Fontenla et al. 2021). However, we found piRNAs and PIWI orthologues in all syndermatans, including the epibiotic and parasitic pararotatorians (Fig. 5a to d, supplementary file S1, Supplementary Material online, “PIWI”). In all syndermatan genomes studied, piRNA-coding sequences were more densely packed than expected by chance, suggestive of their representation in piRNA clusters such as in *Drosophila* and mammalian genomes (Chuma and Nakano 2013; Wierzbicki and Kofler 2023). The *P. laevis* genome assembly exhibited the least compact clustering of piRNA loci (Fig. 5c and e). Moreover, we noticed that piRNAs lacked the ping-pong signature in the bdelloid *A. vaga*, while being retained in the other syndermatan representatives. Furthermore, there was no indication of genes encoding DNA methyltransferase 1 and 3 (*Dnmt1* and *Dnmt3*) in any of the syndermatans analyzed (Fig. 5d). Also, the gene coding for RNA-dependent RNA polymerase (*Rdrp*) was absent in pararotatorians, whereas the monogonont (*Brachionus koreanus*) and bdelloid included (*A. vaga*) had multiple copies. Curiously, there was evidence for zucchini gene (*Zuc*) presence in the *P. laevis* genome, while no counterparts seemed to exist in the other syndermatans, including the acanthocephalan *N. agilis*.

## Discussion

A number of phylogenetic and genomics studies revolutionized our understanding of parasite evolution by largely resolving their relationships to free-living organisms (Aboobaker and Blaxter 2003; Helm et al. 2012; Tsai et al. 2013; Hahn et al. 2014; Foox and Siddall 2015; Sarkies et al. 2015; Lu et al. 2017, 2019; Brabec et al. 2023). Collectively, these investigations demonstrated that morphological reduction is common in the evolution of parasitic life forms. Such simplification may be accompanied by the loss of regulatory microRNAs, which have been proposed to be central drivers of organismic complexity (Sempere et al. 2006; Tanzer and Stadler 2006; Grimson et al. 2008; Heimberg et al. 2008; Peterson et al. 2009; Wheeler et al. 2009; Christodoulou et al. 2010; Berezikov 2011; Fromm et al. 2015; Alberti et al. 2018; Deline et al. 2018; Zolotarov et al. 2022; Fromm 2024; Clarke et al. 2025).

We tested this prediction in helminths of the taxon Syndermata, in which lifestyles range from free-living via epibiotic to endoparasitic (Fontaneto and De Smet 2015; Herlyn 2021). Combining novel short noncoding sequencing data with protein-coding transcriptome, genomic, and morphological data, we provide strong evidence that morphological reduction in parasite evolution is accompanied by loss of microRNAs and protein-coding genes. Loss levels in the epibiotic seisonid and both endoparasitic acanthocephalans were reminiscent of previous reports on BUSCO losses in endoparasitic horsehair worms, Nematomorpha (Cunha et al. 2023). However, with ∼40% missing metazoan BUSCO genes in *Seison* and Acanthocephala, the extent of losses is highest for all animals studied so far. On average, we observed an association in which approximately 425 protein-coding gene losses corresponded to each missing microRNA family, and ∼4 microRNA losses coincided with ∼34 lost metazoan core genes and one lost morphological feature. Of note, limited protein-coding gene losses are common in free-living invertebrate (Steinworth et al. 2023) and vertebrate groups (Emerling et al. 2017) and are considered important in the evolution of metazoans (Albalat and Cañestro 2016; Fernández and Gabaldón 2020; Guijarro-Clarke et al. 2020). In line with this, we detected losses of traits, protein-coding genes, metazoan core genes, and microRNAs on all branches of the syndermatan tree, whereby the extent on the lineage to acanthocephalans was strongest. This supports that genomic regression of parasites is a strong underlying and hierarchical process (see Jackson 2015; Adams et al. 2020) and not just sporadic or mosaic (Tsai et al. 2013; Hahn et al. 2014; Zarowiecki and Berriman 2015; International Helminth Genomes Consortium 2019).

Our results complement previous evidence for a significant role of microRNA losses in parasite evolution in neodermatan platyhelminths (Fromm et al. 2013). Thus, both, parasitic syndermatans and neodermatans, have lost the majority of their microRNA genes present in their free-living ancestors (contra Jackson 2015). However, the extent of losses of widely conserved microRNAs as observed here in seisonid (67%) and acanthocephalan (up to 85%) syndermatans clearly exceeds the corresponding maximum observed in parasitic flatworms (50%) (Fromm et al. 2013). With only 12 microRNA genes in total, the acanthocephalan *P. laevis* has the lowest number of microRNAs reported for any macroscopic bilaterian animal to date. As mentioned above, these counts do not reflect differential contiguity levels of the syndermatan genomes studied, and neither did we uncover additional seisonid or acanthocephalan microRNAs in raw small RNA sequencing reads or unassembled genomic read data. Also, we noticed gains of novel microRNAs in all syndermatan species, although to less extent in the parasites. This general pattern unlikely reflects lower contiguity of the pararotatorian genome assemblies as they are well above the previously detected cutoff of 1E^+4^ (Umu et al. 2023). Indeed, we found no association between N50 values of genome assemblies and the completeness of microRNA complements, supporting the robustness of our results. Nevertheless, we acknowledge that additional data from future high-quality genomes may further refine these observations.

Six novel microRNA families might further substantiate syndermatan monophyly, although this postulate requires their subsequent partial loss within pararotatorian evolution. Moreover, numbers of shared losses (9 losses for Syndermata, 2 additional losses in Hemirotifera, and 16 further losses in Pararotatoria) accord with the most recent phylogenetic analysis (e.g. Vasilikopoulos et al. 2024; Luo et al. 2025). In any case, the hierarchical pattern of microRNA loss clearly supports a “degressive” evolution scenario, and it follows Dollo's law of the irreversibility of evolution (Dollo 1893). Despite the extreme extent of microRNA losses, it is still possible to taxonomically diagnose acanthocephalans based on their minimal retention of microRNAs as syndermatans as they possess one eumetazoan microRNA family (MIR-10), one bilaterian microRNA (LET-7), and two protostome microRNA families (BANTAM and MIR-750), as well as one microRNA family shared only with the other rotifers (Fig. 1) (see also Fromm 2024). It is curious that the acanthocephalans each retain slightly different conserved microRNA complements, which opens up for the possibility that a very strong selective force is acting to (i) remove microRNAs and (ii) retain particular ones in each species. Whether or not the latter could be related to their biology (or hosts) remains to be studied.

Gene Ontology enrichment analysis of metazoan core genes missing in host-bound seisonid and acanthocephalan species revealed a strong enrichment of regulatory terms. This further supports a gene regulatory decline in the pararotatorian stem line through microRNA loss (Fig. 3). Again, loss patterns of protein-coding genes in general and of metazoan core genes in particular did not match with N50 values or genome assembly sizes (Fig. 2a). Previous studies mapping messenger RNA sequencing reads on syndermatan genomes (Mauer et al. 2020, 2021; Hagemann et al. 2024) showed mapping percentages around 90% indicating high completeness and low absence for protein-coding genes further supporting the completeness of these genomes.

Thus, false negatives are unlikely the reason for our findings. Instead, a scenario emerges according to which microRNAs went lost upon the loss of their targets, the protein-coding genes. Strikingly, the loss of 400 proteins per 1 microRNA seems a reasonable ratio, as similar ratios were suggested as potential target numbers for microRNAs (Friedman et al. 2009). However, such a connection may not account for the loss of all microRNAs identified as missing here. This is because individual microRNAs may have even more functional target mRNAs (Liu and Wang 2019). Thus, sufficient other—or newly added—targets may still have been present in syndermatan evolution, despite the loss of individual ones. We wish to investigate this in the future, when high-quality transcriptomic annotations for protein-coding genes, across many species, might be available. Nevertheless, reductions in syndermatan microRNA repertoires and their likely targets seem to have affected ancestral morphological features as evidenced by strong negative correlations with reductions of evolutionary older traits here referred to as plesiomorphies (Fig. 4). Notably, partial correlation analysis corroborated that the association of morphology and microRNA losses is significant irrespective of coherence levels of the genome assemblies included. Complementary evidence comes from GO analysis of BUSCO metazoan core genes, which we found lacking in host-bound pararotatorian species. In fact, many of these genes would engage in translation repression and mRNA decay (Wu et al. 2009; Bartel 2018), thus corroborating less need for expression regulation. This might reflect either reduced regulation of preserved protein-coding genes or loss of some of the targets.

We additionally observed limited gain of novel microRNAs in each syndermatan species. It is tempting to speculate that they might play a role in the differentiation of morphological novelties in the single lineages of Syndermata, although we did not reliably detect their potential target mRNAs. Targets might also be recruited from newly evolved gene products. Either way, it will probably remain that Monogononta, Bdelloidea, and Seisonidea are mainly characterized by retained plesiomorphies from the basic pattern of Syndermata and Gnathifera. In fact, we had intensely searched for morphological apomorphies for all branches of the syndermatan tree, consulting a broad body of literature (for references, see legend to Fig. 4). It seems, however, that the acanthocephalan stem line accumulated the highest number of morphological novelties, in addition to the most morphological reductions. Even if some evolutionary novelties of Acanthocephala, e.g. concerning the tegument or the attachment apparatus, were merged, Acanthocephala would persistently appear to be the most derived taxon within Syndermata. Considerable morphological and developmental reorganization in its stem line is obviously due to the emergence of an endoparasitic two-host cycle (Near et al. 1998; Herlyn 2021). However, this is not a special feature of the Acanthocephala. Rather, a similar trend is found in Platyhelminthes across the phylogenetic lineages of Neodermata, in particular Cestoda, which share an endoparasitic two-host cycle (Deline et al. 2018).

In fact, the list of morphological novelties could be easily extended for acanthocephalans. For example, presomal sensory organs and a peculiar syncytium suspending these have been reported for acanthocephalans only (Dunagan and Miller 1983; Gee 1987; Herlyn et al. 2001; Herlyn 2002). Still, homologues exist for part of the features regarded as acanthocephalan apomorphies in present analysis. For instance, the eponymous hooked attachment organ (proboscis) at the anterior body pole (Meyer 1933; Taraschewski 2014; Herlyn and Taraschewski 2017) probably is a derivative of the anterior body pole, the head so to speak, as maintained in other syndermatans.

Anyway, endowed with many adaptive characters, acanthocephalans have very successfully mastered the challenges of a parasitic lifestyle. This is also evident in frequent reports of new acanthocephalan species upon in-depth investigation of gnathostome vertebrates, which at least occasionally feed on mandibulate arthropods (Amin 2024; Olivera and Campião 2024). Thus, the previous inventory of about 1,200 described species of Acanthocephala (Gibson et al. 2014) is probably far below the actual diversity.

Notably, acanthocephalans and the other syndermatans studied possess the piRNA machinery and express piRNAs. This finding shows that there is no general absence of the piRNA pathway in parasites (Tsai et al. 2013; Fontenla et al. 2021), and other factors beyond parasitism may be the actual drivers of piRNAs loss from certain lineages (Sarkies 2024). It is further worthwhile mentioning that piRNAs retained the characteristic ping-pong signature in most syndermatans studied, except for the bdelloid *A. vaga*. Potentially, the ping-pong pathway that amplifies piRNAs in other species (Brennecke et al. 2007) may not be required in *A. vaga*, considering the duplicated genome of bdelloids (Simion et al. 2021). Here, loss of ping-pong might be compensated by multiple *Rdrp* copies (Nowell et al. 2021; present study) as suggested by ping-pong replacement by RDRP in the nematode *Caenorhabditis*  *elegans* (Batista et al. 2008). Still, absent genes for Dnmt1 and Dnmt3 suggest reduced DNA methylation ability in syndermatans, which herein correspond with other helminth taxa (Hu et al. 2015).

In conclusion, we present compelling evidence for a potential association between the loss of morphological complexity, the loss of protein-coding genes, and the loss of microRNAs in syndermatans. We also observe novel traits, which remain to be analyzed for their genetic basis. For some of these, it has to be clarified, whether they are entirely novel or derived from rearranged structures. Our findings prompt further investigation of the mechanisms whereby loss of microRNAs might either follow morphological reductions mediated by protein-coding mRNA targets or come first. For this, a thorough characterization and quantification of cell types in each species as well as high-quality annotations of protein-coding orthologues between the species and the identification of microRNA target sites within their 3′UTRs would be an exciting next step. The other main regulatory elements of metazoan genomes, transcription factors, or RNA binding proteins have herein not been studied, as their metazoan-wide annotation poses a significant curational challenge, and, thus, their implication in character reduction remains unknown.

The approximation of animal disparity by morphological characters has drawbacks as it often remains unclear whether new characters represent rearranged previously existing features or true novelties, i.e. by the evolution of new cell types. Single cell RNA-Seq methods hold great promise for the future but currently lack standardized methodologies for comparative studies of whole adult animals at scale. Such data will further enable to confirm whether the loss of coding and noncoding genes in parasite evolution is a common feature in all metazoans.

## Materials and Methods

### Sample Collection

About 1,130 males and females of *S. nebaliae* (Seisonidea) were collected in 2018 to 2019 in agreement with the Centre National de la Recherche Scientifique from the surface of opossum shrimps (*Nebalia bipes*) gathered at low tide from rock pools in the tidal flats off Roscoff (France, Brittany). For RNA extraction, 33 males and females of *N. agilis* (Acanthocephala: Eoacanthocephala) were excised from the intestines of thin-lipped mullets (*Chelon ramada*) captured in 2021 in Adriatic coastal waters on behalf of the Po Delta Park administration. Altogether, 35 males and females of *P. laevis* (Acanthocephala: Palaeacanthocephala) were collected from host intestines (common barbel, *Barbus barbus*; European eel, *Anguilla anguilla*) caught by authorized fishermen in a gravel pit near Gimbsheim (Germany) and in the Weser River near Gieselwerder (Germany) in 2006 and 2014, respectively. RNAs from specimen pools of *S. nebaliae* and *N. agilis* were extracted with TriReagent (Invitrogen). The *P. laevis* specimens were distributed over three samples from which RNAs were extracted using TriReagent or miRVana (Thermo Fisher Scientific).

### Genomes

Available genomes for Monogononta (Blommaert et al. 2019; Park et al. 2020; Byeon et al. 2021; Kim et al. 2022, 2018), Bdelloidea (Nowell et al. 2018; Simion et al. 2021), *S. nebaliae* (Mauer et al. 2021), and Acanthocephala (Mauer et al. 2020) and the genomes of outgroup species *Capitella teleta* and *Saccoglossus kowalevskii* were downloaded from GenBank. gDNA reads and nuclear draft genome of *N. agilis* have been reposited at GenBank under BioProject accession PRJNA1126620 and BioSample accession SAMN41948513. Details on generation and key features of the draft genome are given elsewhere (Supplementary Note S2 of Schmidt et al. 2022)

### Next-Generation Sequencing of Short RNA

For *S. nebaliae* one small RNA sequencing dataset, for *N. agilis* two, and for *P. laevis* three small RNA sequencing datasets were generated and sequenced using Illumina by a commercial supplier and the Norwegian Sequencing Centre in Oslo. Small RNA sequencing data are available under BioProject PRJNA1127840 in raw format, and processed and collapsed reads are available on MirGeneDB.org (Clarke et al. 2025).

### Small RNA Processing and MicroRNA Prediction

All 26 genomes were subjected to MirMachine 0.3.0 analyses using protostome models and lophotrochozoan nodes (Umu et al. 2023) to arrive at a set of conserved microRNA families. In the cases of *B. koreanus* (SRR19792092, SRR19792093, SRR19792094) and *A. vaga* (SRR3185495 and SRR3187155), publicly available small RNA-Seq data were analyzed with MirMiner v1. For *S. nebaliae*, *N. agilis*, and *P. laevis*, own small RNA-Seq data were produced (see above). All small RNA-Seq datasets were processed with miRTrace v1 (Kang et al. 2018) (supplementary fig. S1, Supplementary Material online) and analyzed in MirMiner (Wheeler et al. 2009) for MirMachine confirmation and the prediction of novel or species-specific microRNAs. Outputs were manually curated according to the annotation and nomenclature rules described by us (Fromm et al. 2015). Briefly, expression of both arms and 5′ homogeneity giving two nucleotide offsets was expected. Novel microRNAs were blasted to genomes without small RNA-Seq data available. Absence of microRNAs was additionally confirmed using BLAST to all genomes and genomic unassembled DNA reads, respectively. All microRNA complements are available at MirGeneDB.org (Clarke et al. 2025).

### piRNA Prediction and Analyses

Small noncoding RNAs were aligned to the genome using Bowtie (Langmead et al. 2009), to produce SAM files. These were processed using samtools 0.1.18 (Li et al. 2009) and bedtools v2.31.1 (Quinlan and Hall 2010) to generate bed files. The bed files were read into R and processed to determine the length of reads, to test whether there were any sequences with typical length associated with piRNAs (27 nucleotides or longer up to a maximum of 32 nucleotides). Reads with ≥27 nucleotides that mapped to opposite strands were identified, and the overlap between them was tabulated to check for the signature of ping-pong biogenesis, i.e. a ten-nucleotide overlap (Brennecke et al. 2007). To test for the presence of potential piRNA clusters, the number of predicted piRNAs (≥27 nucleotides) in each 2 kb window across the genome was extracted. The windows were sorted by decreasing piRNA density and the cumulative fraction of the total calculated as increasing numbers of windows were added. Clustering of piRNA loci was interpreted as an indication that some regions have very high density, leading to a curve that rapidly plateaus, compared to the random expectation where the piRNA positions were scattered across the genome, and the relationship between the total fraction of piRNAs and the number of windows is more linear (Beltran et al. 2019). From this, the clustering ratio was defined as the number of windows needed to cover 90% of the piRNA sequences when positions were randomized divided by the number of windows needed to cover 90% of piRNA sequences in their real positions. Higher clustering ratio thus indicates that a larger proportion of the piRNAs are in a smaller number of windows. The R code for the analysis is available at GitHub: https://github.com/SarkiesLab/piRNA_rotifer.

### BUSCO and Functional Enrichment

All genomes were analyzed with BUSCO v5.4.3 using the Metazoa node (954 genes) (Manni et al. 2021). The IDs were used to obtain GO terms from OrthoDB v10 database (Kriventseva et al. 2019). We performed an enrichment analysis using the 280 genes missing from both *S. nebaliae* and *N. agilis* as the gene set, and the entire metazoan dataset as the backbone. The overrepresentation analysis was done for all three GO categories (Biological Process, Cellular Component, Molecular Function) using clusterProfiler package (Yu et al. 2012; Wu et al. 2021) in R (Team RC 2013). The computations were performed on resources provided by Sigma2—the National Infrastructure for High-Performance Computing and Data Storage in Norway.

### Morphological Character Analyses

We created a binary matrix of morphological characters of Syndermata starting from the phylogenetically broader compilation by Deline et al. (2018), which in turn referred to characters in three text books by Ax (2012, 2013a, 2013b). In Fig. 4a, we have indicated when we have adapted character definitions by Deline et al. We have also added characters to our morphological matrix. References to adaptations and additions are given for each character in the legend to that table (Nicholas and Mercer 1965; Amin 1987; Ahlrichs 1995; Fontaneto and De Smet 2015; Herlyn and Taraschewski 2017; Herlyn 2021). We additionally correlated morphology losses with microRNA losses controlling for the potential effect of assembly contiguity (N50), by partial rank correlation in SPSS 29.0 (IBM).

## Supplementary Material

evaf124_Supplementary_Data

## Data Availability

New small RNA sequencing data are available under BioProject PRJNA1127840. New gDNA sequencing data are available under SAMN41948513.
