# Supplementary figures and images for: Substantial Hierarchical Reductions of Genetic and Morphological Traits in the Evolution of Rotiferan Parasites
Source: Genome Biol Evol. 2025 Jun 19;17(7):evaf124. doi: 10.1093/gbe/evaf124 (PMC12256650; doi:10.1093/gbe/evaf124)

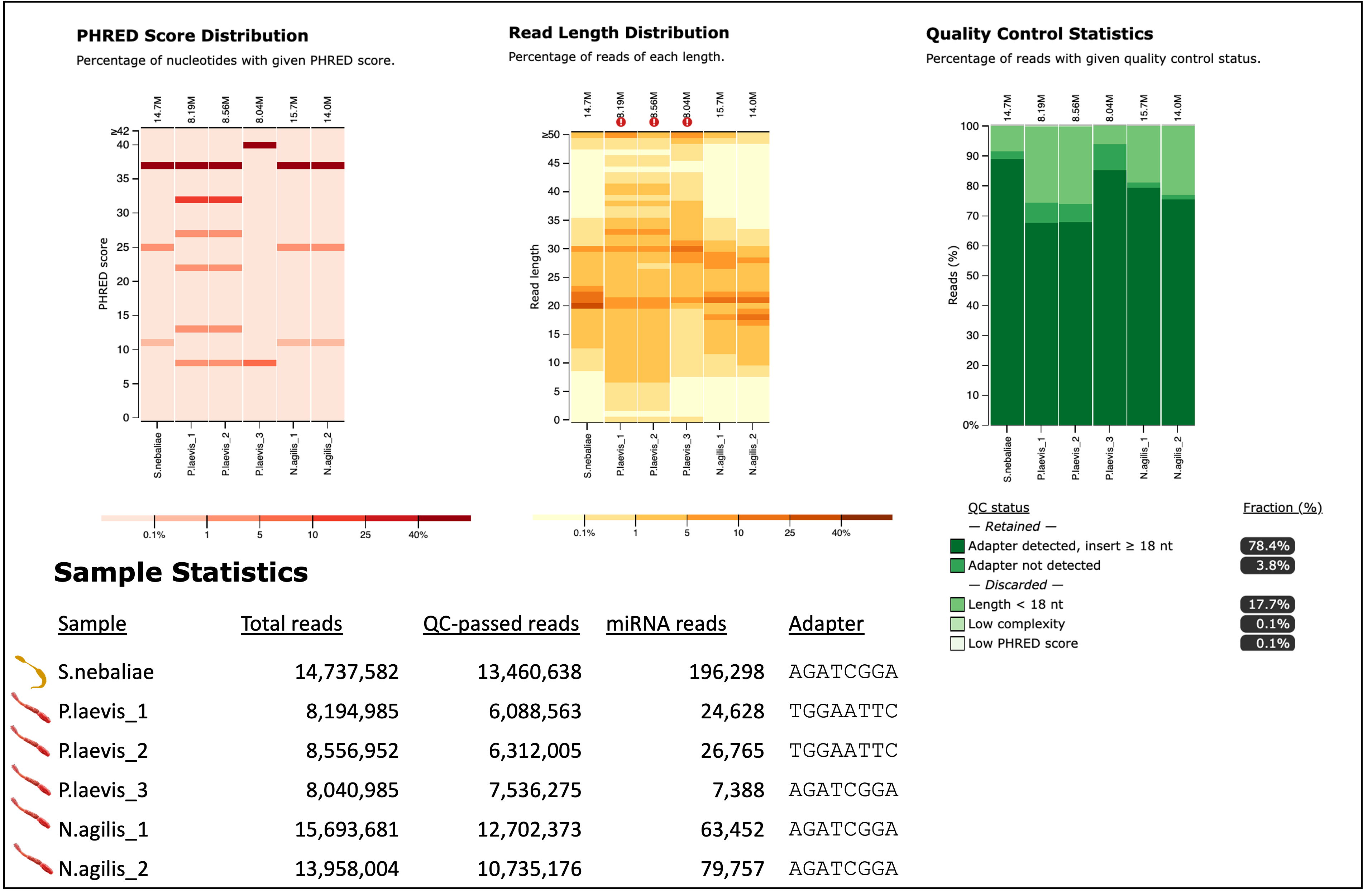

Supplement: evaf124_Supplementary_Data [file evaf124_supplementary_data.zip › Supplementary_Figure1.png]
